# Pathogenesis and Surgical Treatment of Dextro-Transposition of the Great Arteries (D-TGA): Part II

**DOI:** 10.3390/jcm13164823

**Published:** 2024-08-15

**Authors:** Marek Zubrzycki, Rene Schramm, Angelika Costard-Jäckle, Michiel Morshuis, Jan F. Gummert, Maria Zubrzycka

**Affiliations:** 1 Department of Surgery for Congenital Heart Defects, Heart and Diabetes Center NRW, University Hospital, Ruhr-University Bochum, Georgstr. 11, 32545 Bad Oeynhausen, Germany; mzubrzycki@hdz-nrw.de; 2 Clinic for Thoracic and Cardiovascular Surgery, Heart and Diabetes Center NRW, University Hospital, Ruhr-University Bochum, Georgstr. 11, 32545 Bad Oeynhausen, Germany; rschramm@hdz-nrw.de (R.S.); ajaeckle@hdz-nrw.de (A.C.-J.); mmorshuis@hdz-nrw.de (M.M.); jgummert@hdz-nrw.de (J.F.G.); 3 Department of Clinical Physiology, Faculty of Medicine, Medical University of Lodz, Mazowiecka 6/8, 92-215 Lodz, Poland

**Keywords:** transposition of the great artery, etiology, diagnosis, operation procedures and their sequelae

## Abstract

Dextro-transposition of the great arteries (D-TGA) is the second most common cyanotic heart disease, accounting for 5–7% of all congenital heart defects (CHDs). It is characterized by ventriculoarterial (VA) connection discordance, atrioventricular (AV) concordance, and a parallel relationship with D-TGA. As a result, the pulmonary and systemic circulations are separated [the morphological right ventricle (RV) is connected to the aorta and the morphological left ventricle (LV) is connected to the pulmonary artery]. This anomaly is included in the group of developmental disorders of embryonic heart conotruncal irregularities, and their pathogenesis is multifactorial. The anomaly’s development is influenced by genetic, epigenetic, and environmental factors. It can occur either as an isolated anomaly, or in association with other cardiac defects. The typical concomitant cardiac anomalies that may occur in patients with D-TGA include ventriculoseptal defects, patent ductus arteriosus, left ventricular outflow tract obstruction (LVOTO), mitral and tricuspid valve abnormalities, and coronary artery variations. Correction of the defect during infancy is the preferred treatment for D-TGA. Balloon atrial septostomy (BAS) is necessary prior to the operation. The recommended surgical correction methods include arterial switch operation (ASO) and atrial switch operation (AtrSR), as well as the Rastelli and Nikaidoh procedures. The most common postoperative complications include coronary artery stenosis, neoaortic root dilation, neoaortic insufficiency and neopulmonic stenosis, right ventricular (RV) outflow tract obstruction (RVOTO), left ventricular (LV) dysfunction, arrhythmias, and heart failure. Early diagnosis and treatment of D-TGA is paramount to the prognosis of the patient. Improved surgical techniques have made it possible for patients with D-TGA to survive into adulthood.

## 1. Introduction

The most common form of transposition of the great arteries (TGA) is referred to as dextro-TGA (D-TGA) or d-loop TGA. It is the most common cyanotic congenital heart defect (CHD) lesion, and presents in neonates with a frequency of 5–7% [1,2].

D-TGA is a congenital conotruncal abnormality characterized by discordant connections between the ventricles and the great arteries: the aorta arises from the morphologic right ventricle (RV) and the pulmonary artery (PA) arises from the morphologic left ventricle (LV), with preserved atrioventricular concordance (i.e., the LV is connected to the left atrium—LA and PA—whereas the RV is connected to the right atrium—RA and the aorta) [3,4].

Due to such an arrangement, there are two parallel circulations with impaired oxygenation of the peripheral organs. In systemic circulation, deoxygenated blood from the superior and inferior vena cava drains into the RA, RV, and the aorta, whereas in pulmonary circulation, oxygenated blood from the pulmonary veins drains into the left atrium (LA), LV, and PA. The mixing of blood can occur via an atrial or ventricular septal defect, patent ductus arteriosus (DA), or through collateral bronchopulmonary circulation.

The precise etiology of D-TGA has not been fully determined and its pathogenesis is multifactorial and controversial, especially because D-TGA is difficult to reproduce with animal models. The embryological mechanism of D-TGA has traditionally been explained by two main theories. The “extracardiac theory” is based on abnormal spiralization of the aorto-pulmonary septum during cardiac development [5,6]. D-TGA occurs when the conotruncal septum fails to follow its spiral course and forms in a linear orientation instead. The “infundibular theory” suggests that D-TGA is caused by abnormal resorption or the underdevelopment of the subpulmonary conus, with a persistence of the subaortic conus which results in the aorta being placed above the anterior right ventricle [7,8,9].

Many factors, both genetic and environmental, influence the formation of D-TGA. Several genes associated with the pathogenesis of D-TGA, located on different chromosomes, have been identified to date [10,11,12,13]. D-TGA is usually considered to have a low risk of familial recurrence; however, multiple mutations in laterality genes, confirming a pathogenetic relation between TGA and heterotaxy, have been found in some families [14,15,16]. Other studies have postulated the influence of associated risk factors, such as gestational diabetes mellitus [17,18], maternal exposure to pesticides [19], maternal use of drugs during the first trimester [20], a respiratory tract infection during pregnancy, or exposure to ionizing radiation [21], as well as in the case of in vitro fertilization [22,23,24,25].

The clinical features of D-TGA are solely dependent on the degree of mixing between the parallel circuits. Most patients present with signs and symptoms manifested during the neonatal period (first 30 days of life). The typical clinical manifestations of D-TGA include cyanosis, tachypnea, and a murmur resulting from a ventriculoseptal defect (VSD) (pansystolic and prominent at the lower left sternal border) or from pulmonic stenosis (systolic ejection murmur at the upper left sternal border) [26]. Antenatally, D-TGA is difficult to detect on fetal ultrasound due to the absence of differences in ventricle size [27]. Postnatally, when cardiac disease is suspected based on clinical examination, echocardiography is performed, which will reveal the aberrant origins of the aorta and pulmonary trunk as well as any associated intracardiac defects.

It can occur as an isolated anomaly or in association with other cardiac defects. The typical cardiac anomalies that may accompany D-TGA include ventriculoseptal defects, patent ductus arteriosus allowing the combination of oxygenated and deoxygenated blood flow as well as left ventricular outflow tract obstruction (LVOTO), mitral and tricuspid valve abnormalities, and coronary artery variations [28,29,30].

Correction of the defect during infancy is the preferred treatment of D-TGA. Balloon atrial septostomy (BAS) is needed preoperatively in the case of restrictive atrial-level communication leading to cyanosis. Unfortunately, without surgical correction, preoperative mortality in neonates approximates 30% within the first week of life and up to 90% within the first year [31]. The perioperative mortality of D-TGA has improved considerably since the introduction of surgical correction. The initial palliative surgery for D-TGA was atrial switch repair (AtrSR), performed using the Mustard or Senning method, which provided physiological correction. The repair imposes a discordant atrioventricular connection on the existing discordant ventriculoarterial connection to create double discordance [31,32]. Therefore, with AtrSR operations, the anatomical LV becomes the pulmonic ventricle and the RV becomes the systemic ventricle. The high incidence of late-term complications due to this procedure fueled the continued search for the anatomical correction of D-TGA to restore the normal ventricular connections of the great arteries. The arterial switch operation (ASO) was performed by Jatene in 1975 [33]. In ASO, the distal main pulmonary artery and the distal ascending aorta are transected and then anastomosed to their respective ventricles, with the relocation of the coronary arteries to the neoaorta. The ASO has been used instead of the AtrSR operation since the 1980s and is now the standard surgical correction for D-TGA. Definitive surgical procedures for D-TGA include AtrSR, ASO (recommended as the procedure of choice), and the Rastelli and Nikaidoh procedures. As more and more patients who have undergone ASO are living into adulthood, late complications of this procedure have become more evident. The most common postoperative complications include coronary artery stenosis, neoaortic root dilation, neoaortic insufficiency, neopulmonic stenosis, right ventricular outflow tract obstruction (RVOTO), arrhythmias, and LV dysfunction [32,34,35,36,37,38,39,40]. Advances in surgical and medical management have resulted in D-TGA patients surviving into adulthood. The prognosis for patients with D-TGA is generally excellent following surgical correction. The current survival rates are greater than 90%. The ASO has the best long-term survival and functional outcome. Some studies report a >95% survival rate at fifteen to twenty-five years following the discharge from hospital [41,42]. However, neither of these operations is without consequence and they require long-term follow-up.

## 2. Definition of D-TGA and Its Historical Outline

The transposition of the great arteries (TGA) was first described by the British pathologist Mathew Baillie in 1797 in the book *The Morbid Anatomy of Some of the Most Important Parts of the Human Body* [43]. However, the term “transposition of the great arteries” was first used as late as 1814, by the English anatomist John Farre [44]. This means that the aorta and pulmonary trunk were placed (positio) incorrectly across (trans) the ventricular septum (the aorta from the right ventricle and the pulmonary trunk from the left ventricle) [45].

According to Clark’s classification, TGA was considered an anomaly of ectomesenchymal tissue migration and is classically considered to be a conotruncal defect [46]. D-TGA is denoted as the “{S,D,D}” cardiotype in the van Praagh three-letter notation system, where S denotes “situs solitus,” the first letter D denotes ventricular looping (d-looped), and the second letter D denotes the relationship of the great arteries (d-transposed).

There are two main types of TGA, i.e., complete transposition or dextro-transposition of the great arteries (D-TGA), commonly referred to as d-loop TGA, which involves a complete inversion of the great vessels, and congenitally corrected transposition (ccTGA), commonly referred to as l-loop or L-TGA, where the ventricles are inverted instead [40,47,48,49]. In D-TGA, the aortic valve is positioned at the front and on the right side of the pulmonary valve; in L-TGA, the aortic valve is located on the left of the pulmonary valve.

Additionally, cases with complete D-TGA were previously classified as “simple” (isolated) or “complex” [50,51,52]. Simple D-TGA has no associated cardiac anomalies and is more common, while the complex form of D-TGA coexists with other cardiac malformations [52].

**Figure 1:**
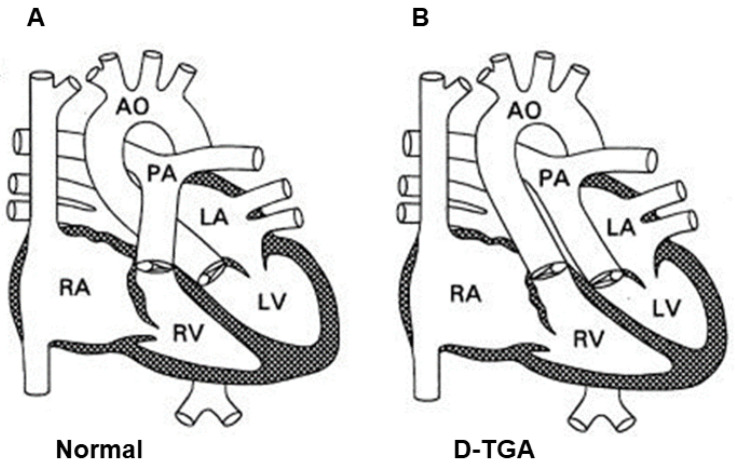
Diagrams of the normal heart (**A**) and D-TGA (**B**). In the normal heart, the pulmonary artery arises from the right ventricle, and the aorta arises from the left ventricle. In D-TGA, due to a complete inversion of the great vessels, the aorta incorrectly arises from the right ventricle and the pulmonary artery incorrectly arises from the left ventricle, whereas the ventricles are normally connected. RA: right atrium; RV: right ventricle; PA: pulmonary artery; LA: left atrium; LV: left ventricle; AO: aorta. This figure was modified and reproduced with permission from Goldmuntz et al. [13].

The most common form of TGA is referred to as dextro-TGA (D-TGA). D-TGA is characterized by discordant connections between the ventricles and great arteries, with the aorta originating from the right ventricle (RV) and the pulmonary artery (PA) originating from the left ventricle (LV) [53] (Figure 1). As a result, an independent pulmonary circuit and systemic circuit are formed. The hemodynamic consequence of these combined connections is that systemic and pulmonary circulations function “in parallel” rather than “in series” [54,55].

In the systemic circuit, deoxygenated blood returns to the right atrium, passing through the tricuspid valve, and is then forced back into systemic circulation by the contraction of the right ventricle and passage into the aberrantly developed aorta. The second circuit is the pulmonary circuit, in which oxygenated blood from the pulmonary veins drains into the left atrium, passes through the mitral valve, and is then forced back into the lungs by contraction of the left ventricle and through the pulmonary arteries. As a result, the necessary oxygenation of systemic blood does not occur and central cyanosis develops. Patients typically present with cyanosis during the first 30 days of life [30]. For a newborn to survive after birth, there must be some kind of communication (connection) between these circulatory systems. Otherwise, the non-oxygenated venous blood is improperly pumped into the systemic circulation and the oxygenated blood from the pulmonary veins is pumped into the pulmonary circulation. Mixing of blood between these two bloodstreams can occur through connections such as patent DA, atrial or ventricular septal defect (ASD and VSD), or through collateral bronchopulmonary circulation. When such a connection does not exist, or is not sufficient to ensure the mixing of blood between the two circuits, a balloon atrial septostomy (BAS) (Rashkind procedure) is performed to facilitate the mixing of blood between the atria [30]. The child’s ability to survive depends on the size of these connections. A lack of sufficient amounts of oxygenated blood in the systemic circulation causes significant hypoxia, which can be fatal in the neonatal period.

## 3. Epidemiology

Dextro-transposition of the great arteries (D-TGA) is a severe congenital heart defect in newborns, representing 5–7% of all congenital heart diseases, corresponding to an incidence of 20 to 30 per 100,000 live births [1,32]. It is predominant in boys, with a male/female sex ratio that varies from 1.5:1 to 3.2:1 [56,57,58]. In the neonatal period, the defect accounts for 25% of cyanotic congenital heart defects. In 10% of D-TGA cases, this cardiac lesion is associated with other non-cardiac malformations [59]. Without surgical treatment, 30% of newborns die in the first week, 50% in the first month, 70% within 6 months, and 90% by the end of the first year of life [60].

With improved diagnostic, medical, and surgical techniques, the overall short-term and mid-term survival rates exceed 90% [61].

## 4. Etiology and Embryological Theories Explaining the Pathogenesis of D-TGA

The etiology for the transposition of the great arteries is unknown; however, it is presumed to be multifactorial. The embryogenesis of TGA is complex and associated with conotruncal defects, the aortopulmonary septum, and disturbances of the bulb rotation that take place in that period [45,46,47,48,49,50,51,52,53,54,55,56,57,58,59,60,61,62]. Currently, there are two main theories which try to explain the embryological mechanisms of TGA development. These are (1) the “extracardiac theory”—an anomaly of the aortopulmonary septum—and (2) the “infundibular theory”—anomalous infundibular rotation.

**Figure 2:**
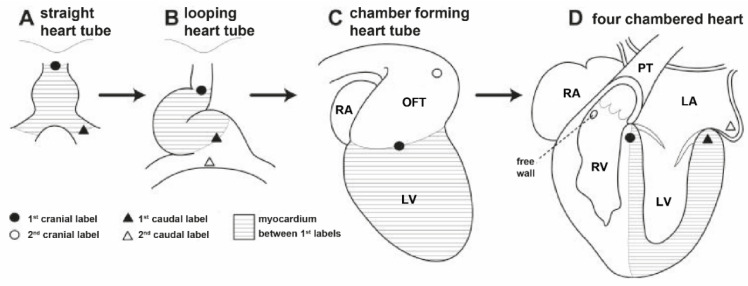
Diagram summarizing the anatomy of the developing heart according to de la Cruz’s theory. (**A**). View of the straight early heart tube. (**B**). View of the looped heart tube. (**C**). View of a chamber-forming heart. (**D**). View of a four-chambered heart, showing the inflow of the left ventricle and the outflow of the right ventricle. LA: left atrium; LV: left ventricle; PT: pulmonary trunk; RA: right atrium; RV: right ventricle. OFT: cardiac outflow tract. This figure was reproduced with permission from van den Berg and Moorman [63].

The first classic theory, proposed by Maria Victoria de la Cruz, focuses on the abnormal spiralization of the aortopulmonary septum. It suggests that in embryogenesis, the aortopulmonary septum does not rotate spirally at the level of the infundibulum. TGA would be the result of a linear rather than spiral development of the aorto-pulmonary septum, which puts the fourth aortic arch (the future aorta) in contact with the anterior muscular conus of the right ventricle (Figure 2) [5,6].

The other theory, originally proposed by Goor and Edwards and corroborated by Anderson and Van Praagh, involves differential conal development [7,8,9]. It suggests that TGA is caused by the lack of the normal, clockwise rotation of the aorta towards the left ventricle [7]. This anomalous infundibular rotation is supposed to be caused by an abnormal resorption or underdevelopment of the subpulmonary cone with an abnormal persistence of the subaortic cone [9]. Persistent subaortic cone and regression of the subpulmonary cone thus leads to aorto-mitral discontinuity and the formation of continuity between the pulmonary trunk and the mitral valve, and displacement of the aorta over the right ventricle and of the pulmonary trunk over the left ventricle. At the same time, the atria of the heart are properly developed, and the visceral–atrial relationship and atrioventricular connections are consistent.

Both of these theories still present important limitations. There are arguments both for and against either of them. The “infundibular theory” seems to explain cases of TGA with ventricular septal defect better, although it is less helpful in explaining cases with intact ventricular septum. On the other hand, the “extracardiac theory” does not account for the great variability of infundibular morphology which characterizes some forms of TGA [64,65]. However, Takahashi et al. demonstrated that spiraling (clockwise) migration of the heart cells is necessary for the proper alignment of the pulmonary outflow tract, so that it may acquire its right-handed spiral pattern [66].

## 5. The Influence of Various Risk Factors on the Etiology of D-TGA

Many factors, both genetic and environmental (biological, physical, and chemical, and maternal diseases), influence the formation of D-TGA [17]. The controversy between environmental factors and genetic causes has been discussed for years. Some studies have postulated associated risk factors, such as gestational diabetes mellitus [17,18], maternal exposure to pesticides (rodenticides and herbicides) [19], and maternal use of antiepileptic and hormonal drugs during the first trimester [20]. The incidence of D-TGA is higher in children of mothers with respiratory tract infections, influenza, and exposure to viruses, and those taking ibuprofen or exposed to ionizing radiation [21], as well as in cases of in vitro fertilization [22]. Exposure to vitamin A, retinol, also has a teratogenic effect on the heart [67]. D-TGA can also be induced by retinoic acid treatments [68] directly involving the TBX2-TGFβ2 pathway [69]. The administration of trans-retinoic acid to pregnant mice at E8.5 of gestation resulted in three-quarters of the fetuses presenting with TGA [68]. In another experiment, mice embryos treated with retinoic acid at day 6.5 presented heterotaxy [68,70]. Cipollone et al. induced TGA by the administration of BMS-189453, a retinoic acid competitive antagonist, to pregnant mice, demonstrating that critical levels of retinoic acid must be present for the normal alignment between the great arteries and the outflow tract [71].

So far, several genes have been identified to be associated with the pathogenesis of D-TGA. They include the thyroid hormone receptor-associated protein-2 gene (MED13L) [72], the nucleocytoplasmic shuttling protein gene (ZIC3) [73], the forkhead activin signal transducer 1 (FOXH1) [74], cryptic family 1 (CFC1) [14], growth differentiation factor 1 (GDF1) [14], the TGF-beta gene (NODAL) [75], and the NK2 homeobox 5 gene (NKX2-5), which is rarely involved in D-TGA pathogenesis [10,76]. In addition, PROSIT240 (also termed THRAP2 or MED13L) mutations have been identified in patients with D-TGA with or without intellectual disability [77]. These genes are located on different chromosomes, and their mutations explain only a few clinical cases. The majority of D-TGA cases remain genetically elusive [12,78]. The chromosomal region 22q11 has also been suggested to be involved in the pathogenesis of D-TGA [79]. There are even several cases of DiGeorge syndrome, with the deletion of chromosome 22q11 [21]. A genomic study carried out in subjects of European descent provides support for a polygenic architecture in D-TGA and identifies a susceptibility locus on chromosome 3p14.3 near *WNT5A* [78]. It has also been found that, due to racial variation and the genetic heterogeneity of TGA, mutations in the MED13L, ZIC3, CFC1, NODAL, FOXH1, GDF1, and NKX2-5 genes are not a common cause of D-TGA in the Chinese population [12]. Mutations in genes involving left–right (LR) asymmetry, such as NODAL, ACTRIIB, and the target FOXH1, have been found in patients with right-sided isomerism, as well as in patients with isolated D-TGA [80].

D-TGA is extremely rarely associated with the most frequent genetic syndromes, such as Turner, Marfan or Down syndrome [81]. In the Saudi Arabian population, D-TGA was not found to be associated with these syndromes at all [82].

However, it can often be found in children with lateralization defects, heterotaxy, and asplenia syndrome (in particular right isomerism) [10,14,83]. *ZIC3*, *CFC1* mutations have been observed in patients with isolated D-TGA and double-outlet right ventricle (DORV), as well as those with sporadic and familial heterotaxy [13,84]. It is important to remember that patients with isolated D-TGA and DORV secondary to an underlying ZIC3 mutation are at an increased risk of having a child with a heart disease [84].

Several studies have used animal models of D-TGA to better understand the mechanisms of the disease. Several genes, such as PITX2, TBX2, *WNT5A*, and CFC1, have been evaluated in mouse models [13,15,69,78] because conotruncal defects with rotational anomalies, including D-TGA, which confirms the importance of the spiral movement of outflow tract, have been demonstrated to occur in PITX2 mutation embryos [5,6,85]. Mice with homozygous mutations of CFC1 develop visceral laterality defects and complex cardiac malformations reminiscent of human heterotaxy syndrome [86,87]. In particular, homozygous mutant mice frequently (i.e., in 82% of cases) have malposition of the great arteries, including D-TGA, as well as other cardiac malformations [86]. It has also been demonstrated that TBX20 attenuates WNT5A expression levels in the developing mouse heart [78]. The above studies highlight the fact that the underlying genetic etiology of D-TGA can be complex. Increasing knowledge about the genetic cause of D-TGA will contribute to improved genetic counseling for individuals and families affected by D-TGA.

## 6. Familial Recurrence of D-TGA

D-TGA is usually considered to have a low risk of familial recurrence, suggesting a polygenic inheritance for the disorder. However, this remains incompletely explored [88,89,90,91,92,93]. In a multicenter English study, the authors reported no familial cases of D-TGA [91], while in an Italian study the recurrence rate in siblings of patients with D-TGA was calculated at 1.7% [90]. Other studies found that the risk of D-TGA recurrence was within the range 0.2–1.4% in siblings [88,94,95]. A recent study demonstrated that in the same families, besides D-TGA {S,D,D} cases, there were first-degree relatives with ccTGA {S,L,L}. In particular, D-TGA recurred in siblings, in first cousins, and in an uncle and nephew [96]. This familial clustering of TGA and ccTGA could be explained by a monogenic inheritance (autosomal dominant or recessive) with a variable phenotypic expression. In some of these families, multiple mutations in laterality genes including Nodal and ZIC3 were found, confirming a pathogenetic relation between D-TGA and heterotaxy [14]. Moreover, in families with heterotaxy, some cases with congenitally corrected TGA were reported and a new gene associated with heterotaxy, CRYPTIC, can present mutations in patients with “isolated” TGA [97].

A study by Alfarhan et al. revealed significant risk factors for the development of D-TGA in the Saudi Arabian population, including gestational diabetes, a family history of congenital cardiac anomalies, and increasing maternal age and parity. These factors increased the risk at least two-fold [82].

## 7. Cardiac Anomalies Associated with D-TGA

Several common cardiac anomalies that can occur in patients with D-TGA include ventricular septal defects (with a certain degree of malalignment of the outlet septum) and LV or RV outflow tract obstructions [28,29,30]. If the outlet septum deviates posteriorly and leftward, sub-pulmonary stenosis is suspected. In contrast, when the outlet septum deviates anteriorly and rightward, subaortic stenosis may occur with a fibrous continuity between the pulmonary and mitral valves. LV outflow tract (LVOT) obstruction is more common in TGA patients with ventricular septal defect. Other congenital heart defects are coronary artery anomalies, right aortic arch, and anomalous venous connections (systemic or pulmonary veins), and mitral and tricuspid valve abnormalities. These anomalies are more frequent in D-TGA patients with ventricular septal defects.

The most common defect accompanying the transposition of the great arterial trunks is a defect in the interventricular septum (20–40%). In most cases, it is a small, hemodynamically insignificant defect that does not require surgical intervention. If the ventricular septal defect is hemodynamically significant, it is usually accompanied by a disproportion between the ascending aorta and the pulmonary artery trunk. In approximately 33% of cases, it is a perimembranous defect located in the area of the membranous septum and tricuspid valve, on the border of its anterior and septal lobes, below the conal septum and above the muscular part of the interventricular septum. In 30% of cases, it is a malalignment type defect. Anterior malalignment is associated with a shift forward and to the right of the conal septum in relation to the muscular septum and may be associated with hypoplasia of the right ventricle, tricuspid valve, or aortic arch, aortic tunnel stenosis, or aortic arch discontinuity. Posterior malalignment means a displacement of the conal septum towards the outflow tract from the left ventricle, with hypoplasia of the pulmonary valve ring and hypoplasia of the pulmonary trunk, which in this variant may be smaller than the ascending aorta. Defects in the muscular part of the septum are described in approximately 27% of cases and defects of the atrioventricular canal type in 5%. The latter type may be accompanied by anomalies of the atrioventricular valve, or disorders of atrioventricular conduction, and less often attachments of the tendon threads of the tricuspid valve on the left side of the interventricular septum (straddling) and hypoplasia of the right ventricle. In 5% of cases, a defect within the conal septum is described. Atrioventricular septal defects (AVSD) are reported very rarely [40,98].

Aortic tunnel stenosis with hypoplasia or rupture of the aortic arch coexists with transposition of the great vessels in approximately 10% of cases and is often associated with subaortic stenosis within the right ventricular outflow tract (RVOTO).

Left ventricular outflow tract stenosis (LVOTO) accompanies 5–25% of cases of transposition of the great arteries and takes the form of a subvalvular or valvular pulmonary trunk. Isolated pulmonary valve stenosis with annular hypoplasia is very rare in D-TGA. Subvalvular LVOTO occurs in approximately 20% of patients with TGA/IVS. In about 10% of them, this stenosis is dynamic in character and is associated with displacement of the interventricular septum caused by higher pressure in the right ventricle. This leads to a characteristic deformation of the interventricular septum towards the low-pressure left ventricle. Such a picture does not occur in newborns with persistent pulmonary hypertension.

In the TGA group with VSD, LVOTO occurs in about 30% of cases and is anatomical or anatomical–dynamic in nature. It is usually a fibrous threshold or ring, and sometimes a tunnel-like or tubular stenosis. The problem may be exacerbated by anomalous mitral valve attachments to the interventricular septum, additional tricuspid valve tissue protruding into the outflow tract through a ventricular septal defect, membranous stenosis, or an aneurysm of the membranous septum. Taking the hemodynamics into account, the presence of critical LVOTO may be the cause of symptoms of small cardiac output and significant hypoxia after ductus arteriosus closure.

D-TGA is isolated in 90% of patients and is rarely associated with extracardiac syndromes or malformations. This congenital heart defect is more common in infants of diabetic mothers [99]. Ferencz et al. demonstrated that extracardiac anomalies had different prevalence in D-TGA (10%, mostly kidney and cerebral anomalies) in comparison with other conotruncal defects (35%), such as tetralogy of Fallot, truncus arteriosus communis, and interrupted aortic arch, which are frequently associated with DiGeorge syndrome and del22q11 [21,100]. Moreover, in that study TGA was found to be more common in males than females, so the authors suggested that TGA should be considered as a CHD etiologically different from other conotruncal defects [100].

## 8. Classification of D-TGA Depending on the Presence of Concomitant Defects

Depending on the presence of concomitant defects, which are decisive for clinical symptoms, the course of treatment, and the surgical technique, we distinguished four different anatomical complexes of complete TGA.

1. TGA with intact interventricular septum (TGA/IVS), the equivalent of the simple form, with accounts for about 36% of all cases. Usually, there is a normal connection at the level of the atria and a patent ductus arteriosus.

2. TGA with ventricular septal defect (TGA/VSD), which accounts for about 29% of all cases. The defect may affect the muscular, membranous (perimembranous), or atrioventricular septum. The ductus arteriosus is unusually wide and the blood flow through the lungs is significantly increased.

3. TGA accompanied by left ventricle outflow tract obstruction (LVOTO) and/or pulmonary trunk valve stenosis. This variant may occur with or without an interventricular defect, usually accompanied by reduced pulmonary flow. It accounts for about 26% of cases. Pulmonary valve atresia and pulmonary artery hypoplasia are very rare in this group.

4. TGA with aortic or subaortic stenosis and often additional stenosis of the right ventricular inflow tract, predominantly with ventricular septal defect and large ductus arteriosus [50,51].

## 9. Clinical Symptoms of D-TGA

Newborns with transposition of the great arteries are usually born at term and are well developed physically. After the delivery, central cyanosis, which does not disappear after the administration of 100% oxygen, is visible. Closure of the ductus arteriosus in the first 24–48 h of life causes rapid deterioration of the baby’s clinical condition. Maintaining patency of the ductus arteriosus causes a difference in oxygen saturation between the upper (preductal) and lower (postductal) parts of the body. If the defect is accompanied by a large DA, an atrial septal defect (ASD), or a large ventricular septal defect (VSD), which allow the blood to mix significantly, the cyanosis may be negligible. In such cases, symptoms of circulatory insufficiency will dominate, exacerbating during the first 2–3 weeks of the child’s life. The child will have shortness of breath, tachypnea, irritability, hepatomegaly, increased second heart tone, and sometimes a heart murmur. Prenatally, D-TGA is difficult to detect on fetal ultrasound due to the absence of differences in the ventricle size [27]. Postnatally, the clinical features of D-TGA are solely dependent on the degree of mixing between the parallel circuits. Most patients present with signs and symptoms during the neonatal period (first 30 days of life). The typical clinical symptoms of TGA include the following:

1. Cyanosis: The degree of cyanosis is dependent on the amount of mixing between the two parallel circuits. The factors affecting intracardiac mixing include the size and presence of an ASD or VSD. Cyanosis is not affected by exertion or supplemental oxygen [101].

2. Tachypnea: Patients usually have a respiratory rate higher than 60 breaths per minute, but without retractions, grunting, or flaring, and appear to feel comfortable.

## 10. Prenatal Detection of D-TGA

The percentage of prenatal diagnoses of D-TGA has been growing for many years but still remains below 50% [102,103]. The essence of prenatal detection of D-TGA is to improve perinatal outcomes and optimize successful transition in the neonatal period [104,105]. Prenatal detection of D-TGA is based on screening and an echocardiographic image examination of the four-chamber fetal heart and evaluation of the fetal heart outflow tracts in three-dimensional (3D), four-dimensional (4D), and spatiotemporal projections, which are crucial to detect D-TGA [106,107]. It is important to identify the key ultrasound markers of D-TGA to improve the prenatal diagnosis and consequently provide perinatal assistance. The presence of two vessels instead of three in the three-vessel tracheal view, a parallel course of D-TGA, and identification of the origin of each D-TGA are the key markers for D-TGA diagnostics [108]. In addition to the classic ultrasound signs, other two-dimensional ultrasound markers such as an abnormal right convexity of the aorta, an I-shaped aorta, and the “boomerang sign” may also be used to diagnose D-TGA in the prenatal period. The diagnosis of D-TGA in fetuses has traditionally been confirmed by identifying the bifurcation of the vessel from the LVOT.

The best method to diagnose D-TGA is to assess the outflow tract based on the following steps: (1) the relationship between the aorta and pulmonary artery should be determined, (2) the anatomic characteristics of the arteries arising from each ventricle should be defined, and (3) the LVOT should be carefully assessed to determine whether an arch or a bifurcation of D-TGA arises from the LV. Fetuses with D-TGA have abnormal outflow tracts showing a parallel relationship with TGA and with the vessel (pulmonary artery) bifurcation arising from the LVOT [108]. These authors highlighted the importance of demonstrating that the pulmonary artery arises from the LV, with the image of its bifurcation resembling the head of a baby bird with an open beak. This sign was described as the “baby bird’s beak image” [108]. Relying solely on first and early second trimester transvaginal fetal echocardiography is not recommended [109].

In addition to fetal echocardiography a few days before delivery, another important tool which could help to differentiate between planned and critical D-TGA is the maternal hyperoxygenation test [110].

The current prenatal classification systems for CHD distinguish between critical D-TGA and planned D-TGA. Critical D-TGA (with a restrictive or closed foramen ovale—FO), is a defect requiring intervention in the first hours and sometimes minutes of the baby’s life to prevent death. Undetected restrictive FO may result in the delivery of a critical newborn that rapidly decompensates before receiving the appropriate cardiac intervention [111]. Prenatally diagnosed D-TGA requires the administration of a prostaglandin infusion after birth and an immediate Rashkind procedure is necessary [111].

Planned D-TGA involves planned surgery after prostaglandin perfusion or the Rashkind procedure later than 24 h after birth [107,112].

Prenatal differentiation of planned D-TGA with sufficient FO from critical D-TGA with restrictive atrial septum is difficult [113], because pulmonary flow will increase during the pregnancy, which may cause the restriction of the FO [114,115]. Changes in the FO in fetuses with D-TGA may occur late in pregnancy [116], and therefore routine assessment of the FO conducted in four-week intervals has been recommended from the time of diagnosis to the time of delivery [111,116,117]. The symptoms of D-TGA depend on the degree of patency of the elements of the fetal circulation (ductus arteriosus, foramen ovale). Also, the ductus arteriosus (DA) should be assessed for abnormal flow in fetuses with D-TGA [118], because the physiology of the flow in DA is different in a D-TGA heart in comparison to normal fetal cardiac physiology [119]. Clinical symptoms appear after a few or several hours and intensify with the closure of the DA [120]. For this reason, it should be mandatory to perform serial fetal echocardiography in the D-TGA, and the assessment of the DA, as well as the FO, just before delivery should be considered extremely important.

The study by van Velzen et al. is one of those which has proven the first-year mortality in prenatally diagnosed D-TGA to be lower than in children without prenatal diagnosis [104]. Children with D-TGA diagnosed prenatally have better early complex cognitive skills, particularly executive function, as compared with those diagnosed postnatally, in whom preoperative acidosis and profound hypoxemia are more common [121]. Prenatal detection, selection between critical and planned D-TGA and appropriate management allow very good, long-term prognosis in fetuses, children, and adults with congenital D-TGA [107].

## 11. Diagnosis of D-TGA

D-TGA diagnosis involves careful history-taking and physical examination of the patient, as well as additional tests.

### 11.1. Heart Auscultation

In the case of complete transposition without ventricular septal defect, the first tone may be loud at the bottom of the sternum, and the second tone is usually narrowly split. Murmurs are not typically present unless there is a small VSD or pulmonic stenosis. A murmur resulting from a VSD will be pansystolic and prominent at the lower left sternal border. Pulmonic valve stenosis causes a systolic ejection murmur at the upper left sternal border [26].

### 11.2. Electrocardiogram (ECG) in D-TGA

In neonates, the electrocardiogram (ECG) may be normal. The P wave tracing is sometimes elevated and sharp. On the 3rd-5th day of life, positive T waves are found in the right ventricular leads and a deviation to the right of the QRS complex axis. They are important symptoms suggestive of right ventricular hypertrophy. The ECG changes rapidly—a picture of right ventricular hypertrophy with a pathological dextrogram develops. A concomitant ventricular septal defect results in the appearance of signs of hypertrophy of both atria and both ventricles.

### 11.3. Chest Radiography (Chest X-ray)

On radiological examination (X-ray) in the postero-anterior projection, attention is drawn to the significantly enlarged silhouette of the heart, in the shape of a large spherical egg with a narrow vascular peduncle (the “egg on a string” appearance); in the lateral projection, the vascular trunk is wide. On the left outline of the heart, a concavity is found in the place of the pulmonary artery trunk that is normally visible. The cavities and peripheral branches of the pulmonary artery present a picture of increased pulmonary flow. The lack of protrusion of the pulmonary artery trunk, and its widened peripheral branches being visible at the same time, as well as the enhanced pulmonary vessel pattern (despite cyanosis), are among the most characteristic radiological features in D-TGA. Enlargement of the heart silhouette is caused by congestive circulatory failure [40].

A chest X-ray and an electrocardiogram may be helpful, but their respective findings are not specific.

### 11.4. Echocardiography of the Heart (ECHO)

It has previously been reported that the prenatal detection rate of D-TGA by fetal echocardiography is low, and it can often be missed [102,103]. However, over time, its detection and diagnosis rates have improved prenatally with advanced three-dimensional (3D), four-dimensional (4D), and spatiotemporal image correlation technology [122,123]. This imaging modality provides accurate morphological and functional assessments of the heart, making it possible to show the specific features of the transposition of the great arteries [28]. In the four-chamber view, atrioventricular concordance is assessed, but the ventriculoarterial discordance is better observed using other incidences. In the five-chamber, parasternal long-axis, or even subcostal view, the vessel arising from the morphologically left ventricle has a posterior course and bifurcates immediately, being identified as the pulmonary trunk. The morphologically right ventricle is related to a vessel that gives rise to the coronary, cephalad, and carotid arteries, i.e., the aorta. The proximal portions of the two arteries run parallel to each other, rather than in the usual cross pattern, giving it a typical and easily recognizable appearance in the parasternal long-axis and subcostal views. In the short-axis view, the pulmonary trunk is usually in a central position, with the aorta situated anteriorly and to the right.

Furthermore, the Doppler study also provides functional information and complements the data provided by the two-dimensional echo. Flow through the arterial duct and septal defects are visualized, indicating the adequacy of mixing between the systemic and pulmonary circulations. Moreover, it is possible to measure gradients through obstructive lesions and to assess the function of the atrioventricular and semilunar valves. Identification of the aortic and pulmonary valves is facilitated by assessing their opening and closing times. The pulmonary valve opens earlier and closes later (unless there is high pulmonary hypertension). An echocardiogram is also useful in detailed assessments of the coronary artery anatomy [124,125] and the exclusion of other developmental defects that may coexist with D-TGA (VSD and LVOTO).

To assess right ventricular function in a modified apical four-chamber view, the following standard echocardiographic parameters should be measured: (1) the SRV diastolic diameter just below the tricuspid valve, (2) the SRV fractional area change (FAC), (3) the tricuspid annular plane systolic excursion (TAPSE), and (4) the s’ velocities at the lateral tricuspid annulus using pulsed-wave tissue Doppler with >140 fps [126]. Conventional color Doppler imaging is used to quantify the severity of tricuspid valve regurgitation. The severity of tricuspid regurgitation is determined based on comprehensive Doppler assessment [127].

Among the novel quantitative echocardiographic techniques, the measurement of regional myocardial strain rate, initially performed using the Doppler technique, can now be conducted using a new method that analyzes movement by tracking speckles (natural acoustic markers)—speckle tracking echocardiography (STE) on a two-dimensional (2D) ultrasound image. The use of STE in the examination of patients with transposition of the great arteries after Mustard/Senning correction has demonstrated that the global longitudinal strain (GLS) of sRV best correlates with its ejection fraction, assessed by cardiac magnetic resonance (CMR) [128]. Recently, it was found that speckle tracking analysis used to assess the fetal heart may lead to the improved detection of fetuses at risk for D-transposition and may also facilitate the prediction of which fetuses with D-transposition will require emergency neonatal BAS [129]. Contrast echocardiography, using echogenic microbubbles, has been used for the assessment of RV myocardial perfusion, with high sensitivity and specificity for detecting heart defects. Contrast with agitated saline bubbles can also be useful to evaluate baffle leaks after the atrial switch operation [130].

Also, new technologies such as cardiovascular three-dimensional (3D) printing currently play a critical role in preoperative evaluation and surgical strategy formulation for complex CHDs. The goal of 3D printing or models is mainly a better analysis of complex anatomies to optimize surgical repair or intervention planning [131]. These 3D modeling and 3D printing technologies are reliable, suitable for the analysis of D-TGA, and complement echocardiography. Further research is ongoing to improve diagnostic tools and techniques for the detection and analysis of D-TGA, because despite technological advances, the detection rate of D-TGA varies.

As echocardiography in experienced hands is a reliable diagnostic tool providing high sensitivity and specificity, the need for catheterization is limited to cases that require the clarification of certain anatomic and hemodynamic aspects not clearly identified by echocardiography.

### 11.5. Cardiac Catheterization and Angiography

Cardiac catheterization and angiography are performed only when echocardiography does not sufficiently reveal the anatomical details of the defect. The assessment of the departure and course of the coronary vessels and the aortic arch is of particular importance. Angiography is rarely used to diagnose D-TGA; however, it is the gold standard for elucidating the origins of the coronary arteries. Cardiac catheterization is routinely used in D-TGA to perform a balloon septostomy in patients with severe cyanosis [132].

Likewise, CT or magnetic resonance imagining (CMR) imaging can also offer additional details of some associated anomalies.

### 11.6. Computed Tomography (CT)

In adults, coronary anatomy can be accurately evaluated using computed tomography angiography (CTA) and CTA is the imaging modality of choice to assess the anomalous aortic origin of coronary arteries from the opposite sinus of Valsalva and their course, the degree of luminal stenosis, the relationship to surrounding structures, and concomitant obstructive coronary artery disease [133].

However, the use of post-ASO computed tomography (CT) is limited in children due to rapid neonatal heart rate and high radiation doses. CT is performed when CMR is contraindicated or expected to generate artifacts [40].

### 11.7. Cardiac Magnetic Resonance (CMR)

In current clinical practice, cardiac magnetic resonance (CMR) is most often performed when echocardiography does not allow for a precise, complete assessment of changes in the heart. Cardiac morphology assessment involves the examination of the myocardium and the structures in its environment. CMR imaging can also provide additional details of some associated lesions. CMR provides comprehensive morphological and functional information, particularly in adults after surgery. Post-ASO CMR is limited by tachycardia and low spatial resolution. Geiger et al. characterized aortic and pulmonary hemodynamics and investigated the correlation with postoperative anatomy in patients with D-TGA on four-dimensional (4D) CMR. They showed increased eddy flow in the anterior position of the pulmonary trunk, asymmetric flow, and increased vortical flow for the anterior of the pulmonary trunk position, and asymmetric flow and increased systolic flow velocity in the pulmonary arteries owing to reduced vascular lumen. However, routine two-dimensional (2D) imaging did not permit the characterization of aortic and pulmonary hemodynamics with full volumetric coverage of the D-TGA cardiovascular system [134].

The only patients who are not eligible for CMR examination include (1) patients with implanted pacemakers, which react to an electromagnetic field; (2) those with a mental illness or disability precluding their cooperation during CMR; (3) pregnant women; and (4) those who did not consent to undergo CMR.

## 12. Prognostic Significance of Markers of Systemic Right Ventricle (sRV) Insufficiency in Patients with D-TGA

Although the atrial switch procedure has been replaced by the arterial switch procedure nowadays, there are still a lot of patients suffering from sequelae after atrial switch, i.e., heart failure (HF) [58]. It has been shown that B-type natriuretic peptide (BNP) and its N-terminal precursor (NT-proBNP) are considered to be potentially the best cardiac biomarkers in the assessment of sRV function and long-term prognosis in TGA [135,136,137,138]. In TGA-RV patients, the NT-proBNP level exceeding 1000 pg/mL seems to be an indicator of worse prognosis [139,140]; therefore, the rise of NT-proBNP levels above this cut-off value, as well as the occurrence of overt HF, are used to define worsening HF. Increased levels of natriuretic peptides are associated with worse clinical outcomes and indicate more advanced disease [138,141,142,143]. As NT-proBNP is a significant prognostic factor for adverse cardiac outcomes in patients with sRV [138,144] and correlates with echocardiography-derived sRV function parameters, its application in elderly D-TGA patients facilitates diagnosis and therapy.

There are data on the use of circulating microRNAs (miRNAs) as novel biomarkers of congenital heart malformations with a systemic right ventricle that is prone to functional impairment. In patients with complex D-TGA, specific miRNAs have been found to be associated with the presence of symptomatic HF, or even to have additional prognostic value to natriuretic peptides. In the study by Lai et al., 11 miRNAs (miR-16, miR-106a, miR-144, miR-18a, miR-25, miR-451, miR-486-3p, miR-486-5p, miR-505, let-7e, and miR-93) were confirmed to be upregulated in D-TGA patients after atrial switch operation [145]. In another study by Abu-Halima et al., it was reported that in patients with D-TGA-RV, miR-183-3p is an independent predictor of worsening HF and thus may be used as an additional biomarker in the risk assessment of these patients [146]. Therefore, in order to characterize the systemic right ventricle (sRV) in D-TGA in terms of the clinical profile, available commercial software dedicated to its assessment is sought.

## 13. Anatomy and Classification of Coronary Arteries in D-TGA

The anatomy of the coronary arteries is of fundamental importance for the planning of surgical treatment, and the technique of their proper transplantation largely determines the early and long-term results of anatomical correction surgery in D-TGA. Variations in coronary anatomy are common and may relate to the position of the coronary ostium relative to the aortic sinus, the angle of coronary take-off, or the course of the coronary arterial branches. The ostia of the coronary vessels are located within the sinuses adjacent to (facing) the pulmonary artery, regardless of the arrangement of the great arterial trunks in relation to each other. If the large vessels are in the front-to-back position, then the coronary vessels are shifted to the right and to the left. If the aorta and pulmonary trunk are located side by side, then the sinuses adjacent to the pulmonary trunk are shifted backwards and forwards, whereas if the aorta is located typically, on the right and anteriorly, then the position of the sinuses adjacent to the pulmonary trunk is left-sided and anterior and right-sided and posterior [147,148,149,150].

From the point of view of surgical technique, the right coronary artery (RCA), which runs in the right atrioventricular groove, the circumflex artery (Cx), running in the left atrioventricular groove, and the left anterior descending artery (LAD), which runs along the interventricular septum on the anterior surface of the heart in the interventricular groove, are of key importance. The most common anatomical variant is the departure of the left coronary artery trunk (LCA) from the left (1st) coronary sinus and its division into LAD and Cx. The right coronary artery departs from the right (2nd) sinus and runs in a normal way. This pattern occurs in approximately 66% of patients with D-TGA. The next most common variant is the departure of the left circumflex coronary artery from the right coronary artery and course posterior to the pulmonary artery, which is seen in about 16% of patients, usually with side-by-side great vessels [148].

The diagnosis and treatment of coronary artery defects have been facilitated by classifications developed for this purpose. Currently, two classification systems, Leiden and Yacoub, are the most widespread.

**Figure 3:**
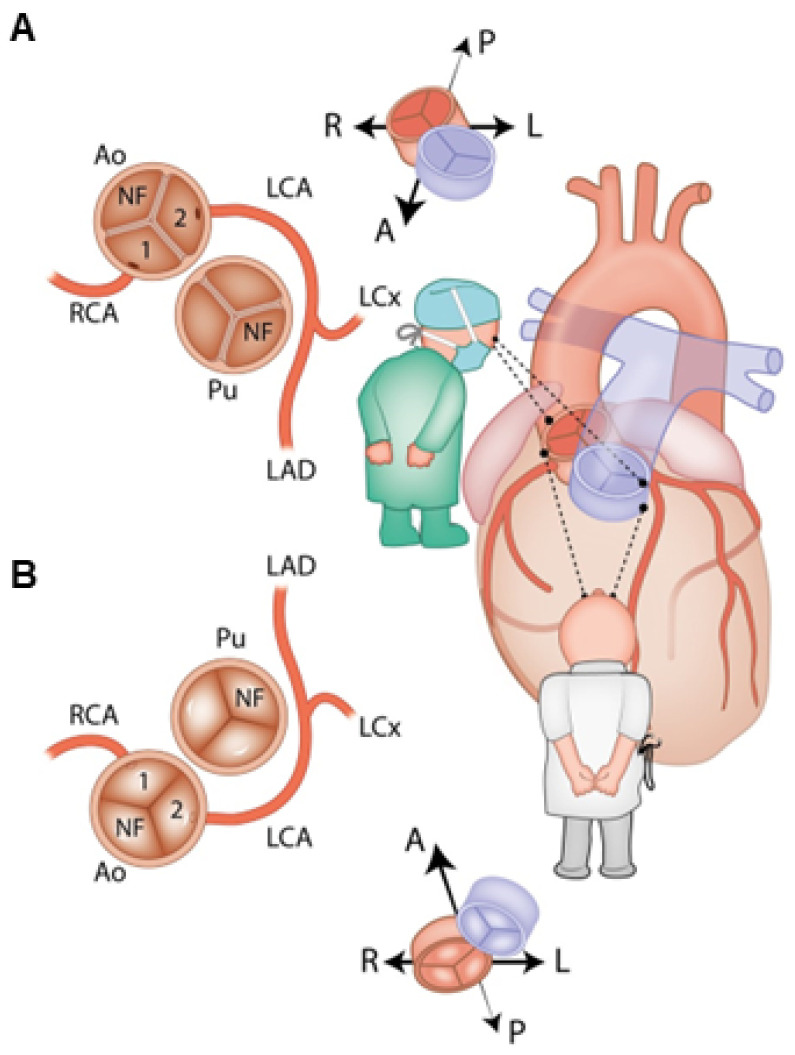
(**A**). Surgical/interventional Leiden convention. (**B**). Imaging Leiden convention. Description in the text. A: anterior; Ao: aorta; L: left; LAD: left anterior descending artery; LCA: left coronary artery; LCx: circumflex artery; NF: non-facing sinus; P: posterior; Pu: pulmonary artery; R: right; RCA: right coronary artery. This figure was reproduced with permission from Koppel et al. [150], under the terms of the Creative Commons Attribution License (CC BY).

In 1986, Quaegebeur developed an alphanumeric classification widely known as the Leiden convention [147,151,152]. This convention describes anatomical variants of the coronary vessels. This coding system is independent of the position of the great arteries relative to each other, such as D-TGA, and considers a surgical/interventional or an imaging perspective (Figure 3) [147].

In the Leiden surgical/interventional convention, coronary anatomy is examined from above, as a surgeon would see it during surgery. The physician sits on the non-facing sinus side, facing the pulmonary valve. In that position, the sinus on the right is sinus 1 and the sinus on the left is sinus 2. Starting from sinus 1, the coronary branches are named in the order that they are encountered when following a counterclockwise rotation (Figure 3A). In the Leiden imaging convention, the physician’s view of the coronary anatomy is from the base of the aorta upward. The physician sits on the non-facing sinus side of the aortic valve, facing outward from the sinus. From this position, the right-hand sinus is again sinus 1, and the left-hand sinus is sinus 2. Following a clockwise rotation, starting at sinus 1, the encountered coronary branches are annotated (Figure 3B) [150].

**Figure 4:**
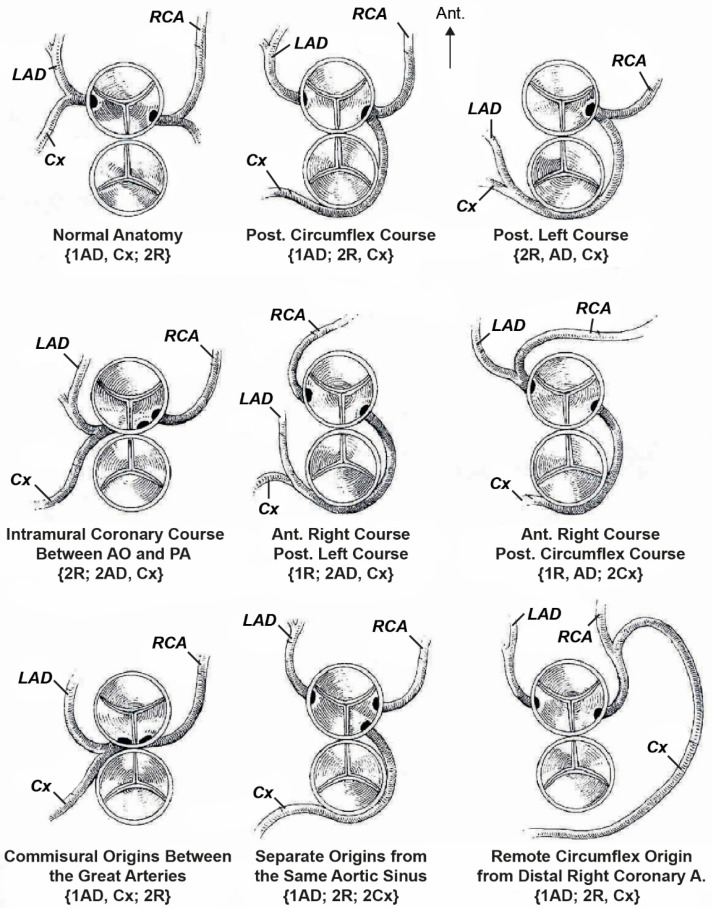
Leiden TGA coronary artery classification. LAD: left anterior descending artery; Cx: circumflex artery; RCA: right coronary artery. The figures are modifications based on the publications by Gittenberger-de Groot et al. [151] and Quaegebeur [152].

The coronary artery pattern was described in a simple system, taking into account the origin of the right coronary artery (RCA; R), the circumflex artery (Cx), and the left anterior descending artery (LAD; L) [152]. According to this convention, the most common (normal) anatomical variant in D-TGA is denoted by the abbreviation {1AD,Cx;2R}, which means the departure of the left coronary artery from the left Valsalva sinus and its division into LAD and Cx; the right coronary artery departs from the right Valsalva sinus (Figure 4).

In 2018, Gittenberger-de Groot et al. proposed the ‘modified Leiden convention’, which also includes the course of the coronary branches around the great arteries. This coding system is applicable to (anomalous) coronary arteries and bicuspid aortic valves irrespective of the position of the great arteries. In this way, coronary anatomy can be uniformly described in all cases, independent of the cardiac anatomy [151]. Over the past decades, the Leiden convention has been widely used among surgeons [153].

Another commonly used classification is based on Yacoub’s studies, where the anatomy of coronary vessels in D-TGA is divided in five subgroups: types A to E [154]. The most common pattern (normal), encountered in nearly two-thirds of the cases, is type A, denoting the departure of LAD and Cx from the left coronary sinus and of the right coronary artery (RCA) from the right coronary sinus. Type B denotes the joint departure of both coronary vessels (single coronary artery) from the left coronary sinus and the RCA course between the aorta and the pulmonary artery trunk. In type C, both coronary vessels depart from their corresponding sinuses, but their orifices are very close to the commissures. Type D is the departure of the LAD from the left coronary sinus, and Cx from the normally draining RCA; the Cx runs in front of the aorta. In type E, the LAD and RCA depart from the left sinus, while the Cx departs from the right one and runs anteriorly to the aorta [148,154,155,156].

The six main coronary artery patterns in D-TGA, as described by some investigators, include four with dual sinus origin of the coronary arteries from the facing sinuses. In the two remaining patterns, there is a single origin from either sinus 1 or sinus 2 (Table 1) [155,157,158,159].

There is also the “French” classification, by Marie Lannelongue, which is based more on the course of the coronary arteries’ vessels rather than on their origin. The main interest of this classification is that the coronary relocation techniques are contingent upon their different courses. Four groups are identified according to the coronary courses and the number of ostia: normal course (two ostia), looping course (two ostia), intramural course (two ostia), and single ostium, miscellaneous course [160,161].

The identification of coronary anatomy prior to surgery is of utmost importance for surgical outcomes. Anomalies of coronary arteries occur in both the origin and distribution, with further anomalies secondary to intramural course in D-TGA [154]. The transfer of the coronary artery origins is a key to a successful ASO. Early mortality is almost always due to difficulty with coronary artery transfer, resulting in myocardial ischemia [37,162]. Obstructed coronary arteries are present in 5–7% of survivors [156,163,164,165] and remain the most common cause of morbidity and mortality following ASO. The incidence of myocardial ischemia, infarction, and death is most prevalent in the first 3 months after ASO.

## 14. Surgical Treatment

The treatment of complete transposition of the great arteries is exclusively surgical and usually involves two stages. The first stage involves a palliative procedure—widening of the foramen ovale or a defect connecting the two atria—and the second involves the correction of the defect. Neonates with high arterial hypoxia, in whom the only connection between the two circulatory systems is the unobstructed foramen ovale, have the worst prognosis and the shortest life. For longer survival of such patients, the oval foramen must be widened as soon as possible in order to increase the degree of mixing of systemic and pulmonary blood and to reduce the pressure in the left atrium.

### Palliative Treatment

The initial aim in the management of the D-TGA-affected newborns is to focus on ensuring adequate oxygenation. The presence of an atrial or a ventricular septal defect that provides satisfactory mixing of the blood permits corrective surgery at a later stage without the need for prior palliative procedures. Nevertheless, this is not the most common situation, and usually a first-line treatment is required.

The first-line treatment involves immediate intravenous prostaglandin E1 (PGE1) infusion, which is used to maintain arterial duct patency leading to an increment in pulmonary blood flow, which increases the pulmonary venous return and left atrial pressure, thus promoting left-to-right flow at the atrial level [166]. Although intercirculatory mixing improves, PGE1 action is often insufficient to assure satisfactory oxygenation of the systemic blood, either in simple or complex transposition.

PGE1 can cause apnea, hypotension, and fever (especially in lower-birth-weight neonates). There may be a false sense of security regarding a preoperative neonate on PGE1 who appears to have “adequate” oxygen saturation. While awaiting ASO, neonates remain at risk for mechanical ventilation, infection, medical errors, paradoxical emboli, increased costs, and a longer hospital stay [167]. The lack of response to PGE1 infusion indicates the need to perform the Rashkind procedure [168].

The method of balloon atrial septosomy (BAS), introduced in 1966 by Rashkind, plays an important role in the preoperative management of babies with D-TGA, especially those without an adequate atrial septal defect, who can develop severe hypoxemia and hemodynamic compromise in the neonatal period. This can be mitigated by urgent BAS. This procedure involves the widening of the foramen ovale or disruption of the atrial septum.

The technique involves inserting a balloon-tipped catheter, through the umbilical vein in the first 48–72 h of the child’s life or, in older newborns, through the femoral vein up to the right atrium and then through the foramen ovale into the left atrium. The balloon is then inflated with contrast medium and pulled back towards the right atrium. A similar maneuver is repeated several times, each time increasing the volume of the balloon. It causes tearing of the atrial septum and creates an opening in it, which will enable effective mixing and improve blood saturation. The result is considered successful when an atrial septal defect with at least 5 mm in diameter and an increased flapping motion of the inferior rim of the atrial septum can be observed and there is an increase in the oxygen saturation [169]. The effectiveness of the procedure is confirmed by a significant increase in oxygen saturation in systemic blood, a decrease in the average pressure in the left atrium as a result of the equalization of pressures between both atria, an increase in systemic pressure, and a significant improvement in the clinical condition.

There have been publications showing that BAS may be associated with vascular trauma, atrial arrhythmias, atrial perforation, and tamponade, with conflicting data regarding an increased risk of stroke. Some reports suggest that embolic brain injury is associated with BAS [170,171], although this has been refuted by others [172,173,174].

However, this procedure is most effective in newborns and infants under three months of age, because they still have a thin, delicate interatrial septum. Balloon septostomy is an effective and safe procedure for creating long-lasting adequate interatrial communications [175].

**Figure 5:**
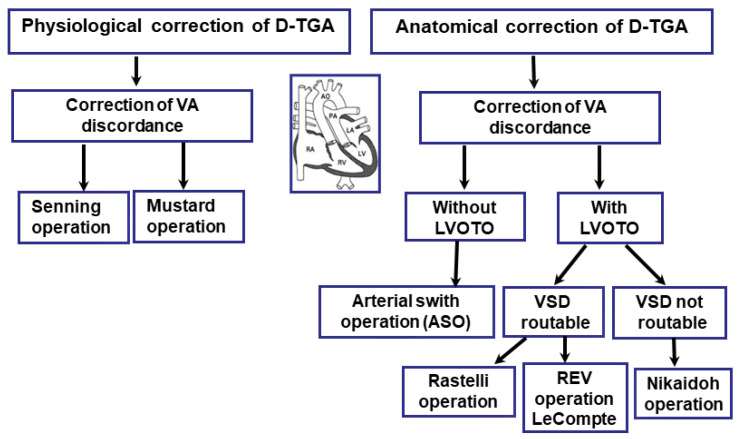
Algorithm for physiological and anatomical correction of D-TGA. D-TGA: dextro-transposition of the great arteries; LVOTO: left ventricular outflow tract obstruction; VSD: ventricular septal defect; ASO: arterial switch operation; REV: réparation à l’étage ventriculaire.

Once the patient is hemodynamically stable, cardiac corrective surgery can be performed. Surgical repair of D-TGA is usually undertaken within the first week of life. There are currently two commonly used surgical procedures for D-TGA: the Jatene arterial switch operation (ASO) and the Rastelli procedure involving the closure of the ventricular septal defect (VSD). There are also other corrective procedures, including the Mustard and Senning procedure, Nakaidoh procedure, and the réparation à l’étage ventriculaire (REV) procedure. However, these are performed less frequently (Figure 5). 

## 15. Anatomical Correction Using the Jatene Method (Arterial Switch Operation, ASO)

**Figure 6:**
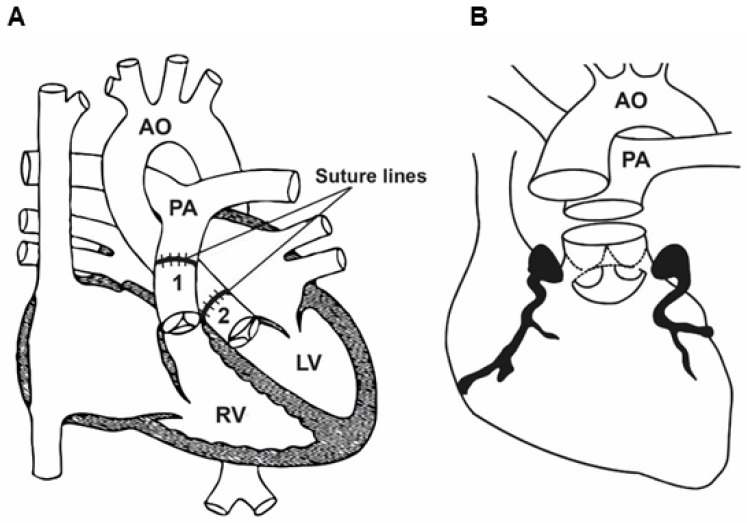
Scheme of arterial switch operation (Jatene procedure). (**A**). The great arteries are transected at the supra-valvar level and reversed in order to create a “normal” anatomical arrangement. 1. Original aortic root—neopulmonary artery root. 2. Original pulmonary artery root—neoaortic root. (**B**). Resection of the coronary arteries with the aortic wall margin surrounding the arterial ostium (marked in black). LV: left ventricle; RV: right ventricle; PA: pulmonary artery; Ao: aorta. The figure was modified based on the publication by Hornung et al. [179].

Arterial switch operation (ASO) has become a standard surgical procedure for the treatment of transposition of the great arteries and some forms of double-outlet right ventricle (DORV), with low mortality and excellent long-term results [176,177] since it was first described by Jatene et al. [33,178]. This method restores the compatibility of ventriculoarterial junctions and consists of supravalvular cutting of the aorta and the pulmonary trunk, and then their displacement and re-anastomosis to their respective (contralateral) ventricles and re-implantation of the coronary arteries in the neo-aorta (Figure 6) [33].

### 15.1. Indications for Surgery

Jatene surgery is performed on newborns of up to 3 weeks of age. In older children, prior preparation of the left ventricle to perform the function of a systemic ventricle is necessary. For this purpose, pulmonary artery banding (PAB) is performed, with the possible formation of a systemic–pulmonary anastomosis (to maintain adequate pulmonary flow). Multiple increased afterload of the left ventricle leads to an increase in ventricular myocardium mass [180]. After this preparatory period, anatomical correction is performed with simultaneous closure of the systemic-pulmonary anastomosis. As a result, the left ventricle acts as the systemic chamber.

### 15.2. Surgical Technique

**Figure 7:**
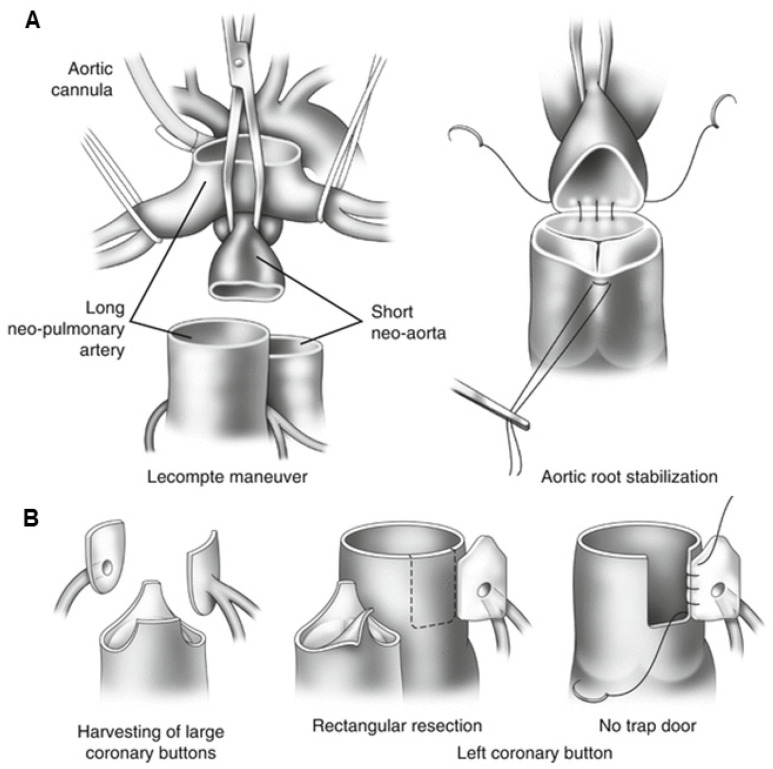
The figures illustrate the surgical technique for arterial switch procedures. (**A**). Replacement of the great arteries with the Lecompte maneuver. (**B**). The harvesting and reimplantation of coronary buttons with normal coronary artery course. Source: The images are based on the publication by Lacour-Gayet [187].

This operation requires the use of extracorporeal circulation and is carried out in deep hypothermia (18 °C). During the surgery, the surgeon will transect both the pulmonary trunk and the aorta and then translocate them to their anatomically correct positions. The procedure requires the transplantation of the coronary arteries into the new aorta (previously the pulmonary trunk) (Figure 7) [178].

After the chest has been opened by a central sternotomy, a pericardial patch is collected and used to reconstruct the pulmonary artery. An arterial cannula is inserted into the aorta at the level of the brachiocephalic trunk. Venous cannulas are placed in the superior and inferior vena cava. After occlusion of the aorta and administration of a cardioplegic solution, the ostia of the coronary vessels (the so-called “coronary buttons”) are excised. The orifices of the coronary vessels are excised together with a U-shaped fragment of the proximal wall of the aorta, and then implanted into the incisions of the proximal part of the pulmonary trunk. After the coronary vessels have been implanted into the proximal part of the pulmonary trunk (the neo-aorta), a Lecompte’s maneuver is performed, as a result of which the pulmonary trunk, located posteriorly in relation to the aorta, is moved forward and takes a position in front of the ascending aorta.

The next stage of anatomical correction surgery is the anastomosis of the aorta with the proximal section of the pulmonary trunk and the pulmonary trunk with the proximal section of the aorta. The defects formed in the wall of the proximal segment of the ascending aorta (after coronary artery resection) are repaired with a patch from the patient’s own (autogenous) pericardium. Techniques with the use of a pulmonary homograft and without the use of any complementary materials (direct anastomosis with appropriate plasty) are also used. The intramural course of the coronary vessel requires cutting off the aortic valve commissure and the excision of a longer fragment of the ascending aortic wall. The commissure of the aortic valve is reconstructed during the reconstruction of the outflow tract to the lungs. Both coronary vessels depart together from a single coronary sinus. In order to avoid tension or twisting of one of the coronary vessels after excision of the common orifice, a flap of the aortic wall is sewn into an incision within the pulmonary trunk (neo-aorta) on the opposite side, and then a roof is made of the autogenous pericardium.

In this context, several techniques for coronary artery implantation have been described, with no clear advantage of one technique over another [181,182]. The most commonly used method is the trap-door coronary transfer technique, which minimizes the rotation angle of a coronary artery by hinging to the neo-aortic root as a trap-door [183]. Another classic closed coronary transfer technique provides optimal geometric configuration for the coronary arteries [181].

Proper coronary artery perfusion and transfer of the coronary artery origins is key to a successful ASO [184]. In a group of patients with closed coronary transfer, a lower risk of the occurrence of late neo-aortic valve regurgitation was observed due to the preservation of the sinotubular junction when this method was used after ASO [184,185]. In a study by Formigari et al., it was reported that the trap-door type of coronary reimplantation is associated with an increased risk for valvular disfunction, possibly because of a distortion of the sinotubular junctional geometry [186]. 

### 15.3. Post-Operative Prognosis of Patients Who Underwent ASO

In large surgical centers, early operative mortality with the ASO approximates 3% [37]. The presence of a concomitant ventricular septal defect (VSD) increases the in-hospital mortality to 6.4% [188]. Overall, 10-year survival rates are reported to reach 88–97% and there is no need for reoperation in approximately 82% of cases [32,165]. At 25 years post-ASO, the conditional survival rate was reported to be 96.7%, with the rate of reintervention being 3.8% [189].

Currently, several studies concerning the ASO and its mid- and long-term outcomes with respect to mortality and morbidity have been published, demonstrating convincing results [32,34,35,36,37,38,39]. Individuals with D-TGA who underwent ASO at a young age have had excellent long-term outcomes so far [38].

Early mortality after ASO is almost always related to coronary artery failure [32,190]. The potential risk for such complications is due to impaired coronary perfusion caused by abnormal patterns of the coronary arteries [153,191,192].

To date, almost 40 years after the initial ASO, a number of long-term post-operative sequelae have been identified [32,36], such as supravalvular pulmonary stenosis, 10%; supravalvular aortic stenosis, 5%; neo-aortic root dilation (nearly 100%); neoaortic regurgitation, 50% (moderate or severe in <10%; asymptomatic coronary artery occlusion, 2–7%; arrhythmias, 2–10%; acute aortic dissection or rupture: incidence rate unknown); and sudden cardiac death, <1% [32,36,38,39,193].

Early sudden cardiac death (SCD) is often associated with technical difficulties during coronary transfer [156]. Ventricular fibrillation and late SCD are usually associated with myocardial ischemia or infarction from coronary obstruction or intimal proliferation [37,41,194,195]. Unfortunately, the incidence of obesity after the ASO is high, making this a potential significant future morbidity. 

Survival into adulthood after ASO is common and the risk of reoperation is low. However, ASO has life-long consequences, some of which may still be unrecognized, requiring ongoing medical surveillance.

## 16. Transposition of the Great Arteries (D-TGA) with Left Ventricular Outflow Tract Obstruction (LVOTO) and Ventricular Septal Defect (VSD)

The cases with concomitant large ventricular septal defects and pulmonary artery outlet stenosis represent a difficult and distinct therapeutic problem. These patients do not have circulatory failure or severe cyanosis, because the mixing of systemic and pulmonary blood at the level of the ventricles is good.

In children with a dynamic left ventricular outflow tract obstruction (LVOTO), it is possible to perform a simple anatomical correction without intervention within the LVOT. A small muscular subvalvular stenosis (usually in children with TGA/IVS) can be corrected from an approach through the pulmonary valve or mitral valve. Such an operation can be postponed and performed in infancy. In the case of a significant impairment of pulmonary flow, a systemic-pulmonary anastomosis may be performed in the first stage, followed by correction at a later age. A significant LVOTO requires the selection of more complex surgical techniques.

In the presence of ventricular septal defect VSD, the choice of surgical technique depends on the location of the VSD. Three parameters are important in the decision-making strategy: (1) the distance of the pulmonary valve from the tricuspid valve, (2) the presence of narrowing of the outflow tract to the lungs, subvalvular and valvular, and (3) the course of the coronary vessels.

## 17. Rastelli Surgical Method

**Figure 8:**
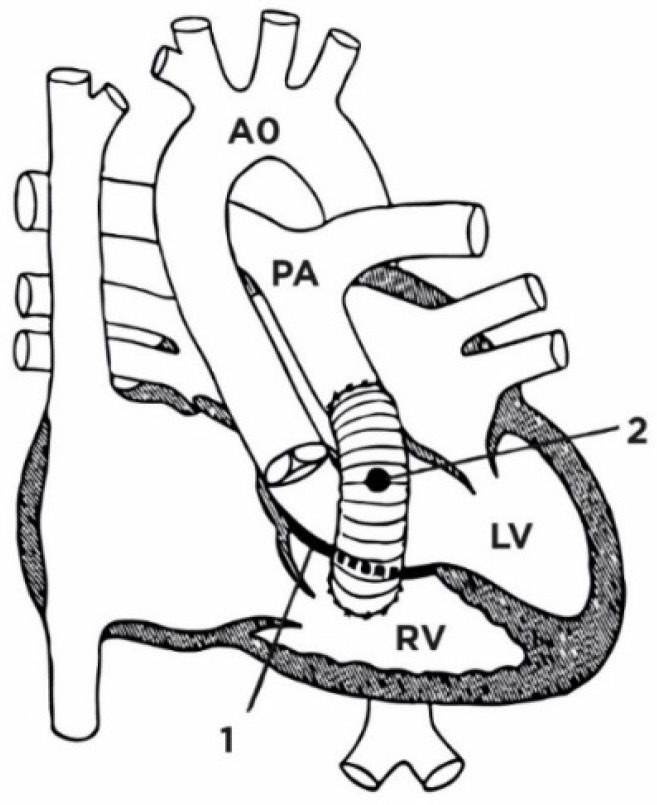
Scheme of the Rastelli operation. 1. The ventricular septal defect is closed with the creation of a left ventricular outflow tract. 2. A right ventricle to pulmonary artery conduit is inserted to bypass the pulmonary stenosis. LV: left ventricle; RV: right ventricle; PA: pulmonary artery; Ao: aorta. This figure was modified based on the publication by Hornung et al. [179].

If the VSD is located closer to the pulmonary valve, and the formation of a non-obstructive tunnel connecting the left ventricle with the aorta is doubtful due to the short distance between the tricuspid valve and the pulmonary artery, the most commonly used surgical procedure is Rastelli surgery (Figure 8). During this procedure, the VSD is closed using a baffle. In this way, oxygenated blood from the left ventricle is directed into the aorta. A conduit is then placed from the right ventricle to the pulmonary artery, thus shunting deoxygenated blood into the pulmonary artery [196,197,198]. 

### 17.1. Indications for Surgery

Rastelli surgery was introduced in 1969 for the treatment of a child with D-TGA, with concomitant significant VSD and left ventricular outflow tract stenosis (LVOTO) [196]. Left ventricular outflow tract stenosis may be caused by a fibrous diaphragm or tunnel, hypertrophy of the ventricular septal muscle, aneurysm of the membranous part of the ventricular septum, an anomaly in the position of the mitral valve, or pulmonary artery valve stenosis. It is assumed that the best period to perform Rastelli surgery is at the age of 4–5 years. The earlier procedure consists of the performance of a balloon septostomy, usually in the first days of life. If cyanosis, hemoglobin, and hematocrit increase in a child with an interventricular defect that meets the conditions for Rastelli surgery, a systemic-pulmonary junction is performed, and often atrial septectomy is performed at the same time. These procedures make it possible to postpone Rastelli surgery until the child is over 2 years of age.

### 17.2. Surgical Technique

The technique involves closing the interventricular defect with a large patch, so that blood from the left ventricle drains to the aortic ostium. The proximal segment of the pulmonary trunk is closed, and the distal segment is connected to the opening in the wall of the right ventricle by means of an artificial vessel, or a homogeneous pulmonary artery, which in most cases will become obstructed.

**Figure 9:**
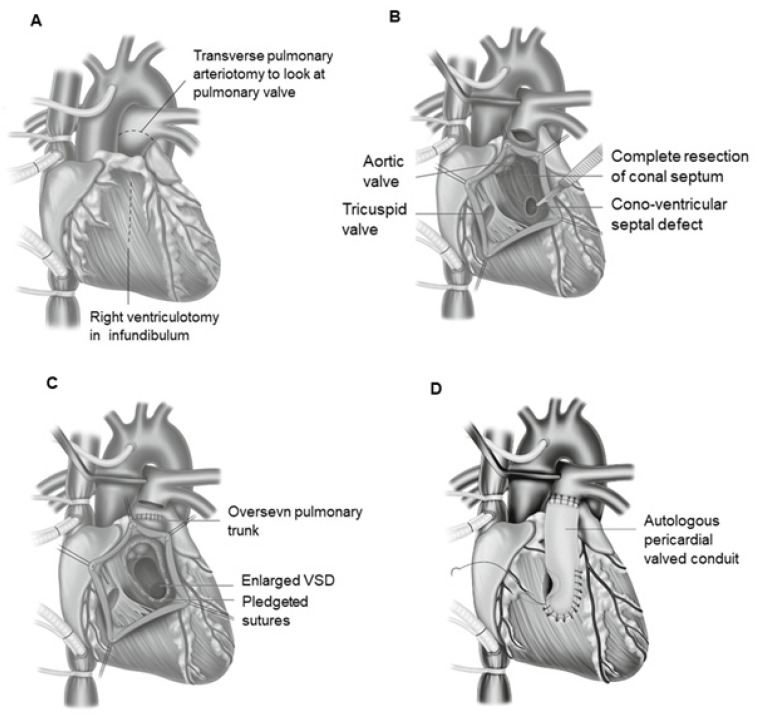
The figures illustrate the Rastelli operation technique. (**A**). The incisions in the pulmonary artery and right ventriclotomy. (**B**). Complete resection of the conal septum. (**C**). Enlargement VSD and the construction of a baffle from the LV to the aorta (ascending). (**D**). RV-to-pulmonary artery continuity is established with the use of a conduit. LV: left ventricle; RV: right ventricle; VSD: ventricular septal defect. Source: The images are based on the publication by Kreutzer [199].

The aim of the operation is to change the blood flow at the level of the ventricles—blood from the left ventricle, through the inner tunnel, flows to the aorta, and blood from the right ventricle, through the implanted valve conduit (usually a homograft), flows to the pulmonary bed. As a result, the left ventricle becomes a systemic ventricle (Figure 9).

## 18. Réparation à l’Étage Ventriculaire (REV)

**Figure 10:**
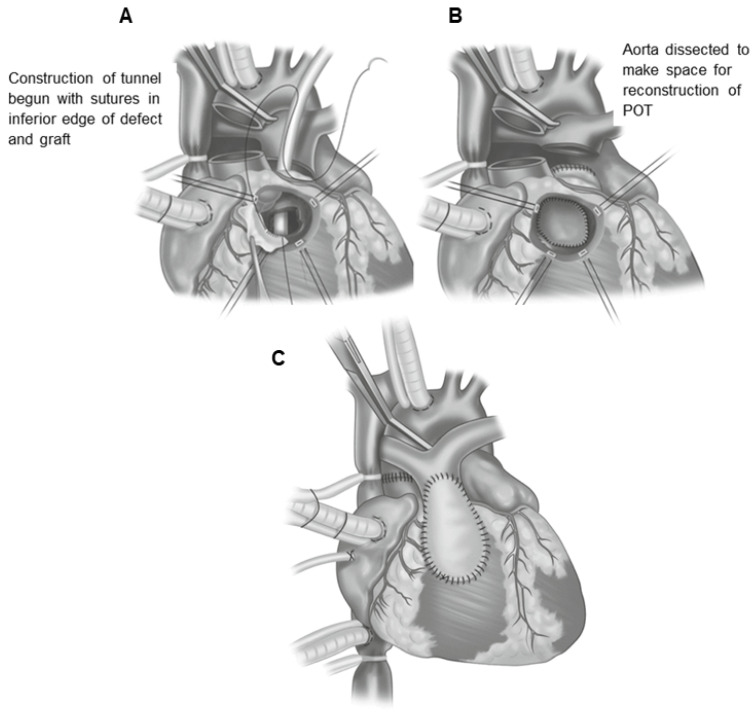
The figures illustrate the réparation à l’étage ventriculaire (REV) operation. (**A**). Construction of the intracardiac tunnel connecting the VSD with the aorta. (**B**). Reconstruction of the ascending aorta and closure of the pulmonary orifice. (**C**). Reconstruction of the pulmonary trunk. RV: right ventricle; VSD: ventricular septal defect. Source: The images are based on the publication by Vouhé and Raisky [202].

An alternative to Rastelli technique is réparation à l’étage ventriculaire (REV) surgery [200,201]. Its essence is the creation of a tunnel connecting the VSD with the aorta (after excision of the cone septum), which also includes the inflow tract into the pulmonary artery (Figure 10). The pulmonary trunk is cut and sutured, and after the transverse incision of the ascending aorta, the pulmonary artery, after careful dissection, is displaced anteriorly (Lecompte procedure) and sutured directly into the ventricle. This combination can be supplemented from the front with additional material. Excision of the cone septum allows for the creation of a tunnel in a straight line, which is not always possible in the case of Rastelli surgery. This technique is less applicable when the main vessels are placed in a side-by-side position. An additional problem may be the compression of the pulmonary artery sewn in under certain pressure on the coronary vessel departing from the anterior circumference of the aorta.

Despite its innovative features, the réparation à l’étage ventriculaire (REV) procedure has not gained significant popularity in the treatment of transposition of the great arteries, ventricular septal defect, pulmonary stenosis, and related anomalies, and thus the Rastelli operation remains the preferred type of repair [203].

## 19. Nikaidoh Surgery

**Figure 11:**
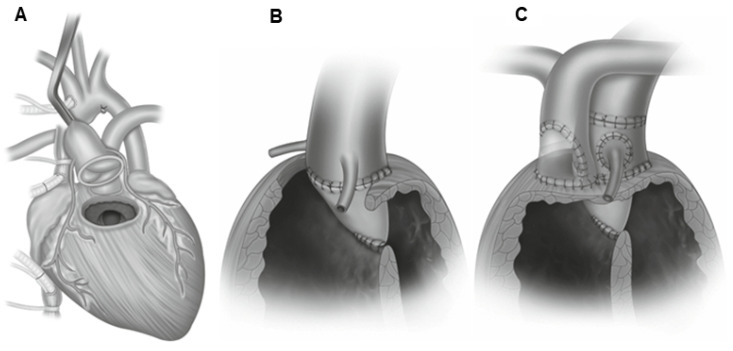
The figures illustrate the Nikaidoh procedure. (**A**). Aortic root harvesting from the RV. (**B**). Closure of the VSD. (**C**). RVOT reconstruction with direct connection of the RV to the PA. RV: right ventricle; PA: pulmonary artery; VSD: ventricular septal defect; RVOT: right ventricle outflow tract. Source: The images are based on the publication by Morell [211].

Bex in 1980 and, later, Nikaidoh in 1984, introduced the concept of aortic root translocation [204,205]. The proposed procedure can be used in patients with D-TGA, LVOTO, and associated non-restrictive or restrictive VSD, especially if inadequate anatomy for an REV or Rastelli surgery is present [205,206]. The essence of this operation is to dissect the aortic root together with the coronary vessels attached to the right ventricle, to incise the interventricular septum, and to enlarge the interventricular defect and the pulmonary valve ring (with resection of the valve leaflets) to suture a patch within the interventricular septum in order to widen the outflow tract from the left ventricle (LVOT), and then to position the aortic root over the left ventricle and anastomose with its left arterial ostium. An aortic autograft is sewn into the outflow tract from the left ventricle, and the ostia of the coronary arteries are sewn into it. The outflow route from the right ventricle is reconstructed using a homograft or a xenograft with a valve [205,207] (Figure 11). The advantages of the Nikaidoh procedure include a straight-line connection between the LV and the aorta, much less reduction in the RV volume, and the placement of an RV-PA conduit in an orthotopic position [208]. However, the procedure is technically demanding and the major disadvantage of this approach is a relatively high rate of reoperations due to right ventricular outflow tract obstruction and pulmonary insufficiency; moreover, unusual coronary patterns may also represent an additional problem [209,210].

An inevitable late complication after the Rastelli or Lecompte operations is the development of outflow obstruction of the left or right ventricle, or of both ventricles, caused by the extracardiac prosthetic conduit as well as the recurrence of LVOTO and arrhythmias [212,213]. As an alternative, the Nikaidoh operation [214] is a novel technique to overcome the shortcomings of the Rastelli and Lecompte procedures; however, it has serious drawbacks concerning coronary insufficiency and subsequent myocardial damage.

The Bex–Nikaidoh procedure was modified in 2004 by Hu et al. to address potential limitations of an RV-PA conduit or a transannular patch [215]. The modification involved preserving the native PV to minimize postoperative pulmonary insufficiency. This could potentially allow the growth of the pulmonary root and therefore decrease the need for reoperation. Other modifications include the Ross–Switch–Konno procedure, as described by Bautista-Hernandez et al. [207].

Despite all these disadvantages, a recent study comparing REV, Rastelli, and Nikaidoh procedures has highlighted the superiority of Nikhaidoh’s approach in obtaining better physiologic cardiac hemodynamics and better long-term survival [216,217,218,219].

## 20. Physiological Correction

However, before anatomical correction surgery, which is now the method of choice for the treatment of D-TGA, became possible, the surgical treatment of this defect had undergone a long evolution related to the development of surgical techniques [168,220,221,222]. The initial palliative surgery for D-TGA was the atrial switch operation (i.e., redirection of the blood flow at the level of the atria), which provided physiological correction and which now has historical significance in the context of this defect. At the end of the last century, arterial switch operation (ASO) replaced atrial redirection according to Senning or Mustard in infants with D-TGA, since improved survival was demonstrated with the new technique [223].

In 1954, Harold Albert described an experimental technique for physiological correction, i.e., redirecting blood flow at the level of the atria in such a way that oxygenated blood flows to the systemic circulation and venous blood to the lungs [224].

The physiological correction technique was developed mainly by two doctors. In 1958, the atrial switch operation was introduced by Dr. Ake Senning of Sweden and shortly thereafter, in 1963, Dr. William Mustard described a comparable procedure.

**Figure 12:**
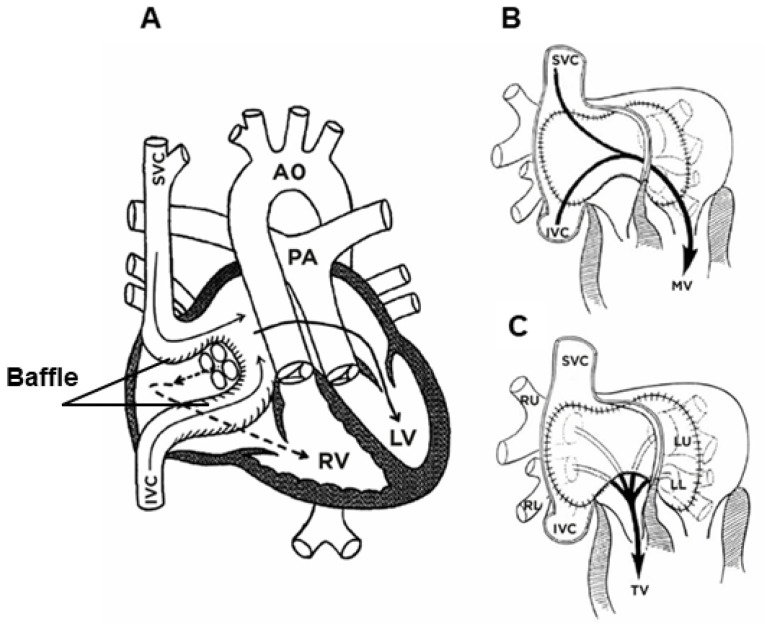
Scheme of the atrial switch operation (Mustard or Senning). (**A**). Neo-atrial baffle diverts blood from vena cava to LV and from pulmonary vein to RV. (**B**). Precise inflow into LV through MV. (**C**). Precise inflow to RV through TV. IVC: inferior vena cava; SVC: superior vena cava; LV: left ventricle; RV: right ventricle; PA: pulmonary artery; Ao: aorta; LL: left lower pulmonary vein; LU: left upper pulmonary vein; MV: mitral valve; RL: right lower pulmonary vein; RU: right upper pulmonary vein; TV: tricuspid valve. This figure was modified based on the publication by Hornung et al. [179].

Both types of correction involve the formation of two tunnels at the level of the atria, bringing blood from the pulmonary veins through the tricuspid valve to the right ventricle and the aorta, respectively, and from both systemic veins, through the mitral valve, to the left ventricle and the pulmonary artery (Figure 12).

The repair imposes a discordant atrioventricular connection on the existing discordant ventriculoarterial connection, thus creating double discordance [31,32]. Therefore, with the Senning and Mustard atrial switch operations, the anatomical LV becomes the pulmonic ventricle and the RV becomes the systemic right ventricle (sRV).

The “redirection” or “crossing” of venous outflows (atrial switch) can be achieved by using only the tissues of both atria and the atrial septum (Senning surgery) for the process of intra-atrial “tunneling” or by using a flap of pericardial tissue or synthetic material dacron (Mustard surgery) instead of the atrial septum. The purpose of the patch is to reverse the direction of blood flowing to the heart from the systemic and pulmonary veins. Thus, the Mustard surgery completely corrects blood flow through the heart without changing the position of the large arteries [225].

### 20.1. Physiological Correction with the Senning Procedure—Surgery Technique

Senning surgery is performed using cardiopulmonary bypass, hypothermia, and cardioplegia. The right atrium is opened anteriorly and in parallel to the sulcus terminalis. Once the boundary ridge and Eustachian valve have been identified, the incision is extended towards these elements. The atrial septum is then cut in such a way that a flap can be formed to make a roof for the outflow of blood from the lungs. Using the produced flap, the ostia of the pulmonary veins are sutured around, and then the suture is extended towards the posterolateral angles of the superior and inferior vena cava. The wall of the left atrium is incised so as to create the widest possible venous outflow, and then a flap of the wall of the right atrium is sutured around the outlet of the superior vena cava, passing to the remnant of the excised atrial septum. The roof of the systemic outlet is formed by suturing the lobe of the wall of the right atrium to the free edge of the Eustachian valve, and if it is not present, then around the ostium of the inferior vena cava and then along the Todar tendon, until it meets the upper suture. The coronary sinus is left in the left atrium. In order to form an outflow from the pulmonary veins, the free wall of the right atrium is anastomosed with the incised wall of the left atrium.

### 20.2. Physiological Correction Using the Mustard Procedure—Surgery Technique

Mustard surgery, like the Senning procedure, requires the use of cardiopulmonary bypass, hypothermia, and cardioplegia. After taking a properly shaped patch from the pericardial sac and connecting the extracorporeal circulation, a longitudinal incision of the right atrium is made between the venae cavae. The atrial septum is widely resected and the coronary sinus is incised, as in the Senning technique. Then, a properly shaped patch of autogenous pericardium is sewn into the atrium, and a tunnel directing the inflow of blood from the vena cava to the mitral valve is created. The right atrium is then closed with a continuous suture.

In patients with a large ventricular septal defect and developed pulmonary hypertension, palliative surgery using the Mustard or Senning technique, leaving the defect or even widening it, is sometimes performed to improve arterial blood saturation. Such treatment usually significantly reduces cyanosis, improving the patient’s general condition, leaving the leak at the level of the ventricles as a safety valve in the event of an increase in pulmonary arterial pressure.

### 20.3. Postoperative Complications after the Mustard and Senning Operations

Initially, the Mustard and Senning atrial switch procedures gained widespread acceptance because they produced good early outcomes and improved survival in patients with D-TGA. Most patients survived into adulthood after Mustard/Senning correction. However, several significant postoperative complications became apparent with increasing age. Complications after AtSR such as systemic baffle obstruction or leak account for most reinterventions, whereas pulmonary baffle obstruction is less common but may cause pulmonary hypertension [226]. During mid-term follow-up, a significant proportion of patients developed arrhythmias and conduction abnormalities [227,228]. Late postoperative complications include right ventricular failure, 15%; moderate–severe tricuspid regurgitation, 20%; bradyarrhythmias requiring pacing, 20%; tachyarrhythmias requiring treatment, 15%; subpulmonary obstruction, 10%; and pulmonary hypertension, 10% [36,229]. These complications were considered to ultimately contribute to sudden cardiac death several decades after an atrial switch procedure [36,230,231,232].

Nevertheless, it is noteworthy that although the overwhelming majority of patients currently qualify for anatomical correction (arterial switch), patients who underwent both Senning and Mustard surgery many years ago still show good health in long-term examinations [233]. The 30-year survival rate of patients discharged from the hospital is 80% [234], whereas the 40-year survival rate reported in cohorts reaches 60–75% [223]. If D-TGA is left untreated, mortality at the end of the first year of life is as high as 90% [235], so this atrial switch offered the possibility of reaching adulthood.

#### 20.3.1. Dysfunction of the Right Ventricle (RV)

There is great concern about the ability of the anatomic right ventricle to sustain systemic circulation in patients with transposition of the great arteries who have undergone the Mustard procedure. As a result of the intra-atrial procedure, the anatomically right ventricle functions as a systemic ventricle (sRV), pumping blood to the main artery, overcoming pressure 3–4 times higher than the pulmonary one, which causes its significant pressure load. Therefore, in order to maintain the correct stroke volume, compensatory dilation occurs. The walls of the anatomically right ventricle are also hypertrophied. Both of these processes lead to an increase in end-diastolic blood pressure [236].

This phenomenon restricts the perfusion of the right coronary artery only to the diastolic phase, which—unlike the left ventricle, under physiological conditions, due to the generation of low pressures—also occurs in the systole. Decreased coronary blood flow and increased pressure load, resulting in an increase in the myocardial demand for oxygen, lead to ischemia. These processes contribute to right ventricular systolic dysfunction in patients after intra-atrial surgery [236]. The diastolic function of the anatomically right systemic ventricle is also impaired. This occurs as a result of myocardial fibrosis as a result of hypoxia, both in the course of preoperative cyanosis and insufficient coronary blood supply. The decreased inflow to the anatomically right ventricle, resulting in its lower output, is also due to the non-physiological direction of blood inflow to the ventricles through artificially created, rigid intra-atrial tunnels. A decrease in the preload on the system ventricle also leads to blood stasis in the venous system.

Recent clinical trials evaluating innovative heart failure medications have shown that three drug classes (mineralocorticoid receptor antagonists [MRAs], angiotensin receptor-neprilysin inhibitors [ARNIs], and sodium/glucose cotransporter 2 [SGLT2] inhibitors) reduce mortality in patients with heart failure with reduced ejection fraction (HFrEF) beyond the conventional therapy consisting of angiotensin-converting enzyme (ACE) inhibitors or angiotensin receptor blockers (ARBs) and β blockers [237,238].

Structural and surgical interventions are targeted for palliation and the prevention of further decompensation in conjunction with pharmacologic, ablative, and device-based therapies for the acute and chronic management of heart failure.

Imaging techniques, including echocardiography, cardiac magnetic resonance imaging, multi-detector computed tomography, and radionuclide ventriculography, focus on the evaluation of anatomy and function as both diagnostic and prognostic tools.

#### 20.3.2. Tricuspid Valve Regurgitation (TR)

The prevalence of tricuspid valve regurgitation (TR) in patients with D-TGA, reported in the literature by various authors, ranges from 2 to 35% [58,239,240]. Severe forms of regurgitation affect 3–10% of that population [179,240]. The development of this complication is the result of the widening of the right ventricle and the subsequent stretching of the tricuspid valve ring, which begins to resemble a saddle. Additionally, the change in the geometry of the anatomically right ventricle subject to overload in terms of pressure and volume (as a result of regurgitation) causes the interventricular septum to become flat or even concave, which changes the spatial arrangement of the subvalvular apparatus, reducing the coaptation of the leaflets. It is also intensified by reduced blood supply, anatomically enlarging the right ventricle and thus contributing to the increasing dilation of the tricuspid ring. The consequence of both phenomena is an increasing impairment of the function of the anatomically right systemic ventricle.

#### 20.3.3. Arrhythmia, Conduction, and Stimulus Generation Disorders

Patients who have undergone intra-atrial D-TGA correction often experience arrhythmias and stimulus disturbances. Venkatesh et al. demonstrated that after 10 years, only 58% of patients have an arrhythmia-undisturbed sinus rhythm [240]. The supraventricular arrhythmia, which dominates in this population, is due to extensive scarring of the atrial myocardium remaining after cardiac surgery, creating conditions for re-entry circulation. This process is also involved in the electrical inhomogeneity of the myocardium of the atria, stretched as a result of the increase in the anatomically right ventricular end-diastolic pressure. The most common form of supraventricular arrhythmia in this population is intra-atrial re-entrant tachycardia (IART) dependent on the tricuspid–venous tunnel [239]. Frequent and prolonged incidents of rapid supraventricular rhythms lead to the formation of so-called tachycardiomyopathy, consequent myocardial ischemia, and ultimately myocardial failure and death [241]. The impairment of the anatomically inferior right ventricular function and aging of the patient exacerbate these phenomena [239]. At the same time, in a vicious circle, exacerbation of heart failure due to dilation of the cardiac chambers and their ischemia may be a substrate for the exacerbation of arrhythmias [58,239]. Atrial fibrillation is rare and usually occurs in older patients [242].

The cause of sudden death in this population has not been fully elucidated. The concept presented by Khairy et al. assumes that the cause of a sudden, lethal decrease in output is a rapid supraventricular arrhythmia conducted by an efficient atrioventricular node, as a result of which the low-yielding systemic ventricle fills and the preload decreases [243]. Hemodynamic decompensation is further exacerbated by sudden hypoxia of the anatomically right systemic ventricle myocardium, working with high frequency, resulting from its inadequate coronary vascularization [233]. However, the significance of ventricular arrhythmia in the mechanism of sudden death is supported by the fact that QT prolongation, which is a risk factor for this arrhythmia, is observed more often in patients who have undergone the surgery [242]. It should also be mentioned that the indications for the primary prevention of sudden cardiac death through the implantation of a cardioverter defibrillator remain unspecified.

Conduction and stimulus disorders are also a frequent complication in adults after intra-atrial D-TGA surgery. These disorders may be caused by congenital anomalies of the sinus node and conductive fibers, their damage during the procedure, or damage to the coronary arteries supplying these anatomical areas. The inevitable degeneration of tissues over time is also important. Only 40–50% of patients operated on according to the Senning method have sinus rhythm after 20 years of follow-up [58,239]. The frequency and extent of conduction and stimulus disorders is influenced by the type of treatment. They are significantly more frequent and advanced after the more extensive procedure performed by the Senning method described above. The method of treatment of sinus node dysfunction and atrioventricular block is pacemaker implantation, which is required by 10–20% of adults with D-TGA at 20 years of follow-up [238,243].

The anatomic right ventricle appears to be unable to sustain systemic circulation at long-term follow-up and the clinical condition of patients after Mustard repair exhibits a decline. These late sequelae are associated with an adverse outcome, hence the importance of timely treatment.

Currently, the management of patients with an sRV is hampered by the lack of evidence-based recommendations because randomized clinical trials are difficult to perform due to the limited population size. However, an individual approach and careful risk stratification, as well as early therapeutic strategies, are crucial to improve long-term outcomes.

## 21. Conclusions

Transposition of the great arteries (D-TGA) is one of the most common and severe congenital pediatric heart defects, arising from an embryological discordance between the aorta and the pulmonary trunk. If it is not treated, it is the leading cause of cardiac death in neonates and infants. The pathogenesis of D-TGA is still largely unknown. In general, D-TGA is not associated with the more common genetic disorders, nor with extracardiac anomalies, whereas it can be found in individuals with lateralization defects, heterotaxy, and asplenia syndrome. The pathological defects in D-TGA cause a detrimental change in cardiac physiology. The initial management of patients with D-TGA focuses on ensuring adequate oxygenation. Prostaglandin E1 administration stabilizes patients by attempting to keep the ductus arteriosus clear and performing a balloon atrial septostomy (BAS). Once the patient is hemodynamically stable, corrective surgery can be performed. Surgical repair of D-TGA is usually undertaken within the first week of life. There are currently two commonly used surgical procedures for D-TGA standard arterial switch operation (ASO): Jatene’s and Rastelli’s procedures are indicated in patients presenting with D-TGA, a large VSD, and pulmonary stenosis. There are also other corrective procedures, including the Mustard and Senning procedure, the Nakaidoh procedure, and the REV procedure. However, these are performed less frequently. Postoperative complications resulting from pathophysiology and surgical intervention and residual defects (residua and sequels) can be the cause of numerous adverse effects, such as right ventricular dysfunction, tricuspid valve regurgitation, supraventricular arrhythmias, or dysfunction of the interatrial and interventricular septa, as well as the less common pulmonary hypertension. Currently, most treated patients live to adulthood, with a 20-year survival of nearly 90%. The most common cause of death is sudden cardiac death, followed by anatomic right ventricular dysfunction. The knowledge of potential complications and the time in which they should be expected allows for treatment that will enable these patients to live into adulthood. Physicians managing patients with TGA should take a multidisciplinary specialist approach to decide which route to pursue and when more advanced treatment options are necessary. Therefore, further research should be conducted to improve surgical techniques, perioperative medical management, surveillance imaging, and the evaluation of long-term post-surgical outcomes.

## Figures and Tables

**Table 1:** The six main coronary artery patterns in D-TGA and their incidence in the literature.

**Type A**	1LCx-2R (70–74%)	**Type D**	1RL-2Cx (2–6%)
**Type B**	1L-2CxR (10–12%)	**Type E**	2LCxR (5–6%)
**Type C**	1R-2LCx (1–2%)	**Type F**	1LCxR (2%)

## Abbreviations

ASD	atrial septal defect
ASO	arterial switch operation
AtrSR	atrial switch repair using the Senning or Mustard methods
AV	atrioventricular
AVSD	atrioventricular septal defects
BAS	balloon atrial septostomy
BNP	brain natriuretic peptide
cc-TGA	congenitally corrected transposition of the great arteries
CHD	congenital heart defect
CMR	cardiac magnetic resonance
CT	computed tomography
CTA	computed tomography angiography
Cx	circumflex artery
DA	ductus arteriosus
D-TGA	dextro-transposition of the great arteries
DORV	double-outlet right ventricle
ECG	electrocardiogram
ECHO	echocardiography
FO	foramen ovale
HF	heart failure
IART	intra-atrial reentrant tachycardia
IVC	inferior vena cava
LAD	anterior descending artery
LCA	left coronary artery trunk
LV	left ventricle
LVOT	left ventricle outflow tract
LVOTO	left ventricle outflow tract obstruction
miRNA	microRNA
MV	mitral valve
NT-pro BNP	N-terminal pro-B-type natriuretic peptide
PA	pulmonary artery
PGE1	prostaglandin E1
RA	right atrium
RCA	right coronary artery
REV	réparation a l’étage ventriculaire
RV	right ventricle
RVOT	right ventricle outflow tract
RVOTO	right ventricular outflow tract obstruction
sRV	systemic right ventricle
SVC	superior vena cava
TGA	transposition of the great arteries
TR	tricuspid valve regurgitation
TV	tricuspid valve
VA	ventriculoarterial
VSD	ventricular septal defect
X-ray	radiological examination
